# Meta-analysis of diagnostic cell-free circulating microRNAs for breast cancer detection

**DOI:** 10.1186/s12885-022-09698-8

**Published:** 2022-06-09

**Authors:** Emir Sehovic, Sara Urru, Giovanna Chiorino, Philipp Doebler

**Affiliations:** 1 Cancer Genomics Lab, Fondazione Edo ed Elvo Tempia, 13900 Biella, Italy; 2 Department of Life Sciences and Systems Biology, University of Turin, 10100 Turin, Italy; 3 Unit of Biostatistics, Epidemiology and Public Health, Department of Cardiac, Thoracic, Vascular Sciences, and Public Health, University of Padova, 35121 Padova, Italy; 4 Department of Statistics, TU Dortmund University, 44227 Dortmund, Germany

**Keywords:** Meta-analysis, Diagnostic, Circulating cell-free, miRNAs, Breast cancer

## Abstract

**Background:**

Breast cancer (BC) is the most frequently diagnosed cancer among women. Numerous studies explored cell-free circulating microRNAs as diagnostic biomarkers of BC. As inconsistent and rarely intersecting microRNA panels have been reported thus far, we aim to evaluate the overall diagnostic performance as well as the sources of heterogeneity between studies.

**Methods:**

Based on the search of three online search engines performed up to March 21^st^ 2022, 56 eligible publications that investigated diagnostic circulating microRNAs by utilizing Real-Time Quantitative Reverse Transcription PCR (qRT-PCR) were obtained. Primary studies’ potential for bias was evaluated with the revised tool for the quality assessment of diagnostic accuracy studies (QUADAS-2). A bivariate generalized linear mixed-effects model was applied to obtain pooled sensitivity and specificity. A novel methodology was utilized in which the sample and study models’ characteristics were analysed to determine the potential preference of studies for sensitivity or specificity.

**Results:**

Pooled sensitivity and specificity of 0.85 [0.81—0.88] and 0.83 [0.79—0.87] were obtained, respectively. Subgroup analysis showed a significantly better performance of multiple (sensitivity: 0.90 [0.86—0.93]; specificity: 0.86 [0.80—0.90]) vs single (sensitivity: 0.82 [0.77—0.86], specificity: 0.83 [0.78—0.87]) microRNA panels and a comparable pooled diagnostic performance between studies using serum (sensitivity: 0.87 [0.81—0.91]; specificity: 0.83 [0.78—0.87]) and plasma (sensitivity: 0.83 [0.77—0.87]; specificity: 0.85 [0.78—0.91]) as specimen type. In addition, based on bivariate and univariate analyses, miRNA(s) based on endogenous normalizers tend to have a higher diagnostic performance than miRNA(s) based on exogenous ones. Moreover, a slight tendency of studies to prefer specificity over sensitivity was observed.

**Conclusions:**

In this study the diagnostic ability of circulating microRNAs to diagnose BC was reaffirmed. Nonetheless, some subgroup analyses showed between-study heterogeneity. Finally, lack of standardization and of result reproducibility remain the biggest issues regarding the diagnostic application of circulating cell-free microRNAs.

**Supplementary Information:**

The online version contains supplementary material available at 10.1186/s12885-022-09698-8.

## Introduction

Breast cancer (BC) is the malignancy with highest incidence and mortality rates among women. In 2020, 2,260 (all ages, in thousands) new BC cases were reported worldwide, with age standardised rates per 100,000 of 47.8 and cumulative risk to age 75 of 5.20% [1]. Moreover, according to the Association of the Nordic Cancer Registries, based on all Nordic countries, the prevalence of BC is around 2% [2]. Considering its high incidence, prevalence and mortality rates, early detection of BC is essential for the prognosis and prevention of the disease. Techniques such as mammography, ultrasonography and sometimes magnetic resonance imaging (MRI) are used for early detection and diagnosis of breast cancer. Nevertheless, mammography and ultrasonography do have some drawbacks in detecting early-stage BC, such as lower sensitivity in younger women or in women with higher breast density [3, 4]. In addition, mammography screens are planned at fixed time intervals, but non-predicted interval cancers may occur between two screens [5]. Moreover, experienced radiologists are required to analyse mammography results [4] as well as to carry out MRI scans, which are also very time-consuming, costly and impractical to be performed routinely. Indeed, there is a general need for accurate BC biomarkers to better guide diagnostic [6] and therapeutic [7] decisions. More specifically, robust minimally invasive diagnostic biomarkers for BC would allow for the improvement in planning of BC screening [8] and its early detection.

Several types of non-invasive biomarkers have been studied in the past years such as polygenic risk scores which involve single nucleotide polymorphisms (SNPs), cell-free DNA, proteins (tumour-associated autoantibodies, carcinoembryonic antigen, carbohydrate antigen, tissue polypeptide-specific antigen, etc.), circulating cell-free or exosomal non-coding RNAs, etc. [9–14]. One type of such biomarkers are cell-free circulating microRNAs (miRNAs). miRNAs are around 22 nucleotide long, single stranded, non-coding RNAs. They play an important role in gene expression regulation as well as epigenetics and cell–cell communication [15]. In their mature form, miRNAs are usually localized in the cytoplasm but can also be exported from the cell [16]. Therefore, some miRNAs are stably found in body fluids such as serum, plasma, saliva or urine as they escape degradation due to their interaction with RNA-binding proteins or exosomes [17]. Diagnostic circulating miRNAs have been studied as biomarkers in different types of cancers [18], including BC [6], and alterations of their levels have been found even before routinely applied diagnostic tools were able to detect tumours [19]. Hence, circulating cell-free miRNAs are potentially more effective in detecting early-stage BC when compared to the other mentioned biomarkers. In addition, they are abundant, very easy to analyse and have a relatively low cost. This hints that circulating miRNAs have a potential for being clinically useful diagnostic biomarkers. Nevertheless, many of the published results were contradictory or non-intersecting as there have been many reported candidate miRNAs or panels of miRNAs but a common significant panel of miRNA(s) as a clinically viable tool was not identified [6]. One reason for this is the lack of experimental and methodological standardization between the studies (e.g. normalizer or specimen type) [6]. Two meta-analyses from 2014 reviewed studies which reported diagnostic circulating miRNAs for BC and concluded that miRNAs have promising diagnostic performance but also stated that a large degree of heterogeneity between the studies exists [20, 21], partly due to the lack of standardization.

In this meta-analysis we seek to include all high-quality evidence on the diagnostic performance of circulating diagnostic miRNA(s) for the detection of BC using any Real-Time Quantitative Reverse Transcription Polymerase Chain Reaction (qRT-PCR) platform. Pooled diagnostic performance, heterogeneity analysis in context of lack of standardization, publication bias as well as general risk of bias in individual studies are the main goals of the study. Unlike the previous meta-analyses conducted on this topic, we have meta-analysed all the reported diagnostic models/miRNAs from each sample, not just one from independent samples within a study. Reports from the same study were considered as dependent (even if they were performed on separate cohorts) and we have taken into account within-study heterogeneity. Moreover, novel methodology is employed for within and between-study preference for sensitivity over specificity based on the case–control ratio, model design and a statistic for the primary study authors’ perceived cost of misdiagnosis.

## Material and methods

### Search strategy and inclusion/exclusion criteria

The methodology was pre-registered in the international database of prospectively registered systematic reviews (PROSPERO; CRD42021229910). The workflow and methodology of the meta-analysis was based on the guidelines of Preferred Reporting Items for Systematic Reviews and Meta-Analyses of Diagnostic Test Accuracy (PRISMA-DTA) [22].

Publications were searched in two databases, PubMed and PubMed Central (NCBI PMC), as well as the Google Scholar search engine. The search was performed up to March 21^st^, 2022. The full search strategy, with the keywords, is documented in the pre-registration. Only peer-reviewed journal articles published in English were considered. Abstracts and other types of publications were excluded. Eligible articles for inclusion were studies which analysed diagnostic performance of circulating cell-free miRNAs in (early stage) breast cancer patients compared to healthy controls or to healthy controls plus patients with benign breast lesions. Therefore, any prognostic studies, studies which analysed exosomal miRNAs, studies which did not have a miRNA model based on qRT-PCR data and studies which did not have a model with healthy controls were excluded. The study designs included in this meta-analysis are retrospective or prospective case–control studies. Studies which included more than 4.5% metastatic (TNM Classification of Malignant Tumours stage IV) breast cancer patients were also excluded. It was also required that the studies report diagnostic performance data (sensitivity, specificity, area under the curve of the receiver operating characteristic (ROC AUC), etc.). Studies from which the frequencies of true positives (TP), false positives (FP), true negatives (TN) and false negatives (FN) could not be directly or indirectly extracted were excluded. In case studies had unclear, but existing, patient data they were included in the analysis, but the authors were contacted for clarification. However, studies which did not specify whether stage IV cases were included and did not specify their number/percentage were excluded from the study if the authors did not reply to our inquiry. In addition, since the Google Scholar search engine was used, we checked whether all article’s journals were peer-reviewed and indexed before inclusion in the full-text eligibility evaluation.

### Data extraction and synthesis

The obtained set of items and research publications obtained from the mentioned search sources were collected as a list in one spreadsheet. All duplicate hits were removed. First the publication type, title and keywords were evaluated by reviewers ES and GC. Then the abstracts of all articles which were not excluded in the initial evaluation were read. In case of any disagreements a third reviewer PD was the arbiter. Afterwards the articles which satisfied inclusion criteria based on screening of abstracts were selected for the full text evaluation, which was performed thoroughly, again by ES and GC, in order to decide on inclusion or exclusion. In all three steps the reasons for exclusion were documented. Lastly, a list of articles fully eligible for this meta-analysis was compiled.

Using the same data extraction protocol and data structure, data from the selected articles was independently extracted by ES and GC. In case disagreements occurred between the two reviewers, PD was the arbiter. From each study the country, bibliometric data (author, year and journal), patients’ average or median age, patients’ breast cancer stage distribution (from stage 0 to stage IV), diagnostic performance data (TP, FP, TN, FN; potentially several miRNA models were reported and if a study had a train as well as test/validation cohort the performance data were extracted only for test/validation cohorts), ROC AUC value(s), normalization method, cut-off value(s), sample size of all groups, miRNA(s) profiled, specimen type, platform information and statistical model information were extracted. In addition, from the reported ROC curves, the q-Point of the ROC (intersection of the anti-diagonal line on the ROC plot with the ROC curve) as well as three other points, aiming for equal distance between them, which were not on the extremities were extracted. As some studies only reported a ROC curve, the q-Point was extracted in order to obtain a uniform performance statistic from all the models. This enabled a complementary analysis because there were more studies which reported a ROC curve than studies with diagnostic performance data. The three additional points were extracted to fit a parametric ROC curve which would then be used for the preference analyses. The extraction of the mentioned points from the ROC graphs was performed using the digitize function from the digitize package in R software [23].

### Risk of bias analysis

All the included studies were evaluated, independently by two reviewers, ES and GC, using the revised tool for Quality Assessment of Diagnostic Accuracy Studies (QUADAS-2) [24] in order to evaluate the potential risks of bias (in four key domains: patient selection; index test; reference standard; flow and timing). The QUADAS-2 was tailored to be more suitable for studies which dealt with diagnostic performance of miRNAs for early BC diagnosis. The main changes were made in Domain 2 (Index test) and Domain 4 (Flow and Timing). For each variable in QUADAS-2, the percentage of agreement between the two reviewers was determined. Discrepancies in coding and/or QUADAS-2 evaluations were resolved by trying to reach a consensus. In case no consensus could be reached, a third reviewer PD was the arbiter.

### Statistical analysis

Primary studies use a wide range of computational methods to obtain estimates of diagnostic performance and ROC-curves, including classification methods like logistic regression and machine learning when the screening result depends on more than one variable. In this paper we will refer to the study level computations as models, even if the computations are relatively simple. By utilizing the diagnostic performance data (TP, TN, FP, FN) of the models, the sensitivity, specificity and diagnostic odds ratio (DOR) were calculated. In addition, other diagnostic performance parameters of the model such as positive likelihood ratio (PLR), negative likelihood ratio (NLR), positive predictive value (PPV), negative predictive value (NPV), accuracy, etc. were calculated. Confidence intervals of PPV and NPV were calculated using the formula from [25] if sensitivity or specificity were equal to 1, otherwise logit transformation from [26] was applied. A formula from [27] was used to calculate the confidence intervals of PLR and NLR.

Descriptive statistics on diagnostic performance data was calculated using the madad function from the mada package in R software [28]. The equality of sensitivities and specificities, as well as the DOR and their confidence intervals were calculated. In addition, the correlation of sensitivities and false positive rates was calculated. Forest plots of sensitivities and specificities, the crosshair and ROC ellipse plots were based on those models labelled as the preferred model by primary study authors or, if no preferred model was specified, on the best performing model (from now on ‘most important model per study’).

To estimate pooled sensitivity and specificity, two bivariate mixed models (in this case referred to the statistical analysis models) were performed: one including all the models and one considering only one model per study. In the first model, random effects on models and studies were added to take into account the between- and within-study variance. In the latter, only the random effect on study was considered, resulting in the bivariate model from [29]. The approach was implemented with the glmer function in the lme4 package [30], recommended by [31], and the SROC was plotted for both models. The analyses were repeated on subgroups to detect possible differences in the performance measures. Subgroups analyses were based on normalizer type, specimen type, miRNA profiles (single or multiple miRNA panel) and presence of stage III and/or stage IV cases (< 4.5% as previously described). In addition, a subgroup analysis was performed on 3 subsets of studies depending on their QUADAS-2 score. Specifically, the score was determined by the number of “low” classifications (indicating a low probability of bias) among the seven key QUADAS-2 questions. The cut-points of the three subsets were set at > 3, > 4 and > 5 “low” classifications.

#### Sensitivity analysis

The outlier analysis was performed on all the models which have reported diagnostic performance data. It was calculated based on the odds ratio. After having calculated the odds ratio for all models, the z-scores were calculated and a cut-off of z-score > 2 was selected for classifying outliers. Influence analysis was performed on both all models as well as the most important model per study. Cook’s distance of the bivariate mixed models was calculated using the influence function from the influence.ME package [32]. The z-scores were calculated on Cook’s distance and models with a z-score > 2 were deemed as influential.

#### Imbalance of proportions

To compare the performance of models with the imbalance of proportions of cases to controls or predicted positive to predicted negative screens, all reported models were divided in 3 groups. The cut-points for imbalance of proportions were set at < 0.7, > 0.7 and < 1.3 and > 1.3. A graphical technique was utilized where the models were plotted on a ROC plane and marked according to the imbalance of proportions group they belonged to.

#### Implicit cost of misdiagnosis

Despite similar accuracy in terms of statistics like the AUC, study level ROC curves can have very different shapes. Assuming authors consciously or intuitively balance the shape of the study level ROC curve in accordance with the primary screening purpose, the study level ROC reflects a preference or compromise between sensitivity and specificity in the context of a population level prevalence. Based on a method of [33], we include two statistics explained subsequently: (i) The shape parameter α that quantifies the (a)symmetry of the study level ROC curve. A value of α = 1 indicates a ROC curve symmetric around the anti-diagonal on ROC space. Low values of α indicate a preference of specificity over sensitivity at the same overall accuracy, while high values lead to a preference of sensitivity over specificity. (ii) The cost parameter c_1_ that is a measure of the (implicit) author perceived cost of a false negative misdiagnosis in relation to the cost of a false positive misdiagnosis. A value of c_1_ = 1 indicates that for the prevalence at hand, authors chose a cut-off value for the primary study’s ROC curve that assumes equal cost of both types of misdiagnosis. Values lower/higher than 1 correspond to lower/higher cost of a false negative case in relation to a false positive case. Detailed explanations of these statistics are in Supplementary Methods (see Additional file 1).

#### Publication bias

The escalc function from the metafor package [34] was used to calculate the effect sizes and sample variances of the models, which were then used to generate a funnel plot. In order to test for publication bias, Egger’s test using the rma.mv function [34] was performed. All statistical analyses were performed in R [35]; script and dataset can be found in the github repository of the project (https://github.com/saraurru/Meta-analysis-of-diagnostic-cell-free-circulating-miRNAs-for-BC-detection).

## Results

**Fig. 1:**
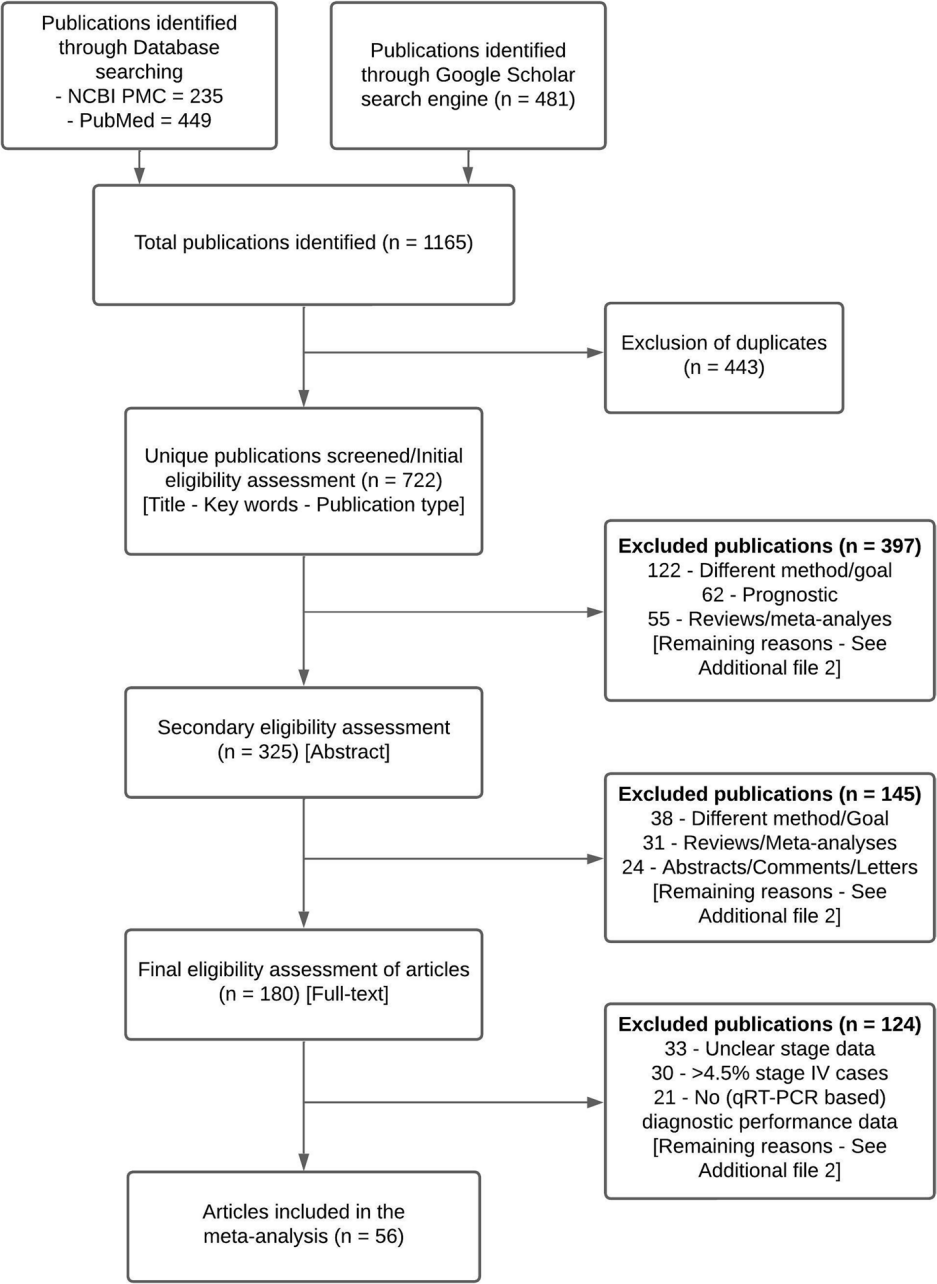
Flow diagram of the selection procedure for the inclusion of studies in the meta-analysis

A total of 1,165 publication hits were obtained after performing a search in two databases (PubMed and NCBI PMC) and the Google scholar search engine (Fig. 1). PubMed and NCBI databases yielded 449 and 235 publications, respectively. The Google Scholar engine yielded 481 hits. After the removal of duplicates (*n* = 443) 722 unique publications were obtained. Type of publication, title and keywords were evaluated in the initial eligibility assessment while the abstract was evaluated in the secondary eligibility assessment. In the initial and secondary eligibility assessment 397 and 145 publications were excluded, respectively. The final, full-text, eligibility evaluation was performed on 180 articles, of which 124 were excluded. Hence, a total of 56 articles remained eligible for the meta-analysis. A generalized summary of the exclusion reasons for all three eligibility evaluation steps is shown in Table 1, while the comprehensive and complete list of reasons and their frequencies are available in Supplementary Table S1 (see Additional file 2).

**Table 1:** Summary of the exclusion reasons for all three eligibility evaluation steps

Reason for exclusion	Number
Abstracts/Comments/Letters	60
Metastatic focus	17
Dubious article/Language/Not found	45
Different method/Goal	162
No performance data	54
Too specific subtype of BC	11
Unclear stage data	33
> 4,5% stage IV samples	31
Review/Meta-analysis	86
Prognostic	68
Not related to BC	3
Exosomal miRNAs	23
Therapeutic	47
Not biomarker focused	26
**Total excluded publications**	**666**

### Included studies

Within the 56 studies which analysed the performance of circulating miRNAs in diagnosis of BC using qRT-PCR, a total of 3,894 cases and 2,948 controls were included. The sample size range of BC patients among the studies was from 15 to 180, while the range of controls was from 10 to 199. The case and control number of each study is based on the model within each study with the largest case/control number. The studies were conducted in 15 different countries: Belgium (*n* = 1), China (*n* = 21), Egypt (*n* = 7), Germany (*n* = 3), Indonesia (*n* = 1), Iran (*n* = 6), Iraq (*n* = 1), Kazakhstan (*n* = 1), Lebanon (*n* = 1), Mexico (*n* = 2), Rwanda (*n* = 1), Singapore (*n* = 1), South Korea (*n* = 2), Spain (*n* = 4), USA (*n* = 3) and 1 included samples from multiple institutions. Hence, 8 studies were conducted in Africa, 34 in Asia, 8 in Europe, 5 in North America and 1 study was multicontinental. Seven of the 56 studies included stage IV breast cancer patients, 4.5% or less of the total cancer patient cohort. The remaining 49 studies did not include any stage IV cases. Ten of the 56 studies did not report diagnostic performance data but reported ROC graphs with AUC values, while 3 studies did not report ROC graphs with AUC values but reported only diagnostic accuracy in terms of sensitivity and specificity. Key information about the included studies can be seen in Table 2.

**Table 2:** General information about the studies included in the meta-analysis. The references of the studies were marked with an asterix symbol in the References section

**Authors**	**Year**	**Country**^a^	**Source**	**Sample size (Healthy controls + benign)**^b^	**Index test (model)**	**Diagnostic Performance**^c^
Swellam et al. [36]	2019	Egypt	Serum	182 (39 + 47)	- miR-21	0.857
					- miR-126	0.998
					- miR-155	0.995
				111 (39 + 47)^d^	- miR-21	0.400/0.930
					- miR-126	0.760/1.000
					- miR-155	0.958/0.965
Zhang et al. [37]	2017	China	Whole Blood	28 (13)	- miR-30b-5p	0.933
					- miR-96-5p	0.769
					- miR-182-5p	0.759
					- miR-374b-5p	0.826
					- miR-942-5p	0.813
Mar-Aguilar et al. [38]	2013	Mexico	Serum	71 (10)	- miR-10b	0.950
					- miR-21	0.950
					- miR-125b	0.950
					- miR-145	0.978
					- miR-155	0.994
					- miR-191	0.794
					- miR-382	0.967
					- miR-145/miR-155/miR-382	0.988
Wu et al. [39]	2012	China	Serum	100 (50)	- miR-222-3p	0.670
Diansyah et al. [40]	2021	Indonesia	Plasma	42 (16)	- miR-21	0.923
Hosseini Mojahed et al. [41]	2020	Iran	Serum	72 (36)	- miR-155	0.890
Pena-Cano et al. [42]	2019	Mexico	Serum	100 (50)	- miR-195-5p	0.882
Kim et al. [43]	2020	South Korea	Plasma	60 (30)	- miR-202	0.950
Heydari et al. [44]	2018	Iran	Serum	80 (40)	- miR-140-3p	0.660
Motamedi et al. [45]	2019	Iran	Plasma	47 (24)	- miR-21	0.828
Swellam et al. [46]	2019	Egypt	Serum	150 (30 + 40)	- miR-17-5p	0.871
					- miR-155	0.993
					- miR-222-3p	0.863
				103 (30 + 40)^d^	- miR-17-5p	1.000/0.757
					- miR-155	0.935/0.944
					- miR-222-3p	1.000/0.786
Matamala et al. [47]	2015	Spain	Plasma	230 (116)	- miR-505-5p	0.721
					- miR-96-5p	0.717
					- miR-125b-5p	0.637
					- miR-21	0.607
Li et al. [48]	2019	China	Plasma	226 (113)	- let-7b-5p/miR-122-5p/miR-146-5p/miR-210-3p/miR-215-5p	0.966
Han et al. [49]	2017	China	Serum	120 (21)	- miR-21	0.788
				71 (21)	- miR-125b	0.559
				120 (21)	- miR-145	0.587
				70 (21)	- miR-155	0.749
				120 (21)	- miR-365	0.795
				70 (21)	- miR-21/miR-155	0.868
				70 (21)	- miR-21/miR-155/miR-365	0.918
				120 (21)	- miR-21/miR-365	0.868
Zhao et al. [50]	2010	USA	Plasma	30 (15)	- let-7c	0.780
				30 (15)	- miR-589	0.850
				20 (10)	- miR-425	0.830
				20 (10)	- let-7d	0.990
Pastor-Navarro et al. [51]	2020	Spain	Serum	90 (45)	- miR-21/miR-205	0.773
					- miR-21	0.771
					- miR-205	0.649
Si et al. [52]	2013	China	Serum	120 (20)	- miR-92a	0.923
					- miR-21	0.933
Freres et al. [53]	2015	Belgium	Plasma	196 (88)	- miR-16/let-7d/miR-103/miR-107/miR-148a/let-7i/miR-19b/miR-22*	0.810
					- miR-16/let-7d/miR-103/miR-181a/miR-107/miR-142-3p/miR-148a/let-7f-1/miR-199a-5p/miR-590-5p/miR-32	0.800
Schrauder et al. [54]	2012	Germany	Whole Blood	48 (24)	- miR-202	0.680
Ng et al. [55]	2013	China	Plasma	120 (50)	- miR-145/miR-451a	0.931
Li et al. [56]	2018	China	Plasma	292 (146)	- miR-106a-3p/miR-106a-5p/miR-20b-5p/miR-92a-5p	0.826
			Serum	298 (148)	- miR-106a-5p/miR-19b-3p/miR-20b-5p/miR-92a-3p	0.965
Shen et al. [57]	2014	USA	Serum	100 (50)	- miR-133a/miR-148b	0.860
Antolin et al. [58]	2015	Spain	Whole Blood	64 (20)	- miR-200c	0.850
				37 (20)	- miR-200c	0.820
Soleimanpour et al. [59]	2019	Iran	Plasma	60 (30)	- miR-21	0.990
					- miR-155	0.920
Nashtahosseini et al. [60]	2021	Iran	Serum	72 (38)	- miR-660-5p	0.774
				62 (38)^d^	- miR-660-5p	0.816
				72 (38)	- miR-210-3p	0.716
				62 (38)^d^	- miR-210-3p	0.652
Han et al. [61]	2020	China	Serum	182 (38)	- miR-1204	0.823
Chen et al. [18]	2016	USA	Plasma	102 (49)	- miR-21	0.613
					- miR-152	0.687
Yu et al. [62]	2018	China	Serum	160 (47)	- miR-21-5p/miR-21-3p/miR-99a-5p	0.895
Zou et al. [63]	2021	China	Serum	246 (122)	- let-7b-5p/miR-106a-5p/miR-16-5p/miR-19a-3p/miR-19b-3p/miR-20a-5p/miR-223-3p/miR-25-3p/miR-425-5p/miR-451a/miR-92a-3p/miR-93-5p	0.956
Fang et al. [64]	2019	China	Plasma	131 (38 + 40)	- miR-324-3p/miR-382-5p/miR-21-3p/miR-324-3p/miR-30a-5p/miR-30e-5p/miR-221-3p/miR-324-3p	0.901
					- miR-324-3p/miR-382-5p/miR-21-3p/miR-324-3p/miR-30a-5p/miR-30e-5p/miR-221-3p/miR-324-3p	0.820
An et al. [65]	2018	China	Serum	109 (24)	- miR-24	0.716
					- miR-103a	0.721
Hu et al. [66]	2012	China	Serum	152 (76)	- miR-16/miR-25/miR-222/miR-324-5p	0.928
Zhang et al. [67]	2015	China	Serum	151 (93)	- miR-205	0.840
Eichelser et al. [68]	2013	Germany	Serum	160 (40)	- miR-34a	0.636
					- miR-93	0.699
					- miR-373	0.879
Wang et al. [69]	2018	China	Serum	102 (44)	- miR-130b-5p/miR-151a-5p/miR-206/miR-222-3p	0.931
					- miR-130b-5p	0.728
					- miR-151a-5p	0.796
					- miR-206	0.861
					- miR-222-3p	0.886
Zhang et al. [70]	2017	China	Plasma	125 (50)	- miR-200c	0.557
					- miR-141	0.582
Feliciano et al. [71]	2020	Spain	Serum	80 (60)	- miR-125b/miR-29c/miR-16/miR-1260/miR-451a	1.000/0.8167
				188 (92)	- miR-125b/miR-29c/miR-16/miR-1260/miR-451a	0.962/0.922
Ibrahim et al. [72]	2020	Egypt	Plasma	50 (20)	- miR-10b	0.730
					- miR-21-3p	0.780
					- miR-181a	0.700
					- miR-145	0.700
Swellam et al. [73]	2021	Egypt	Serum	94 (20 + 30)	- miR-27a	0.818/0.920
Jang et al. [74]	2021	South Korea	Plasma	136 (56)	- miR-1246	0.963
					- miR-206	0.935
					- miR-24	0.965
					- miR-373	0.935
					- miR-1246/miR-206	0.988
					- miR-1246/miR-206/miR-373	0.991
					- miR-1246/miR-206/miR-24/miR-373	0.992
Guo et al. [75]	2020	China	Plasma	79 (40)	- miR-21	0.658
					- miR-1273 g-3p	0.633
Huang et al. [76]	2018	China	Serum	235 (107)	- let-7a	0.683
					- miR-155	0.638
					- miR-574-5p	0.891
Ashirbkekov et al. [77]	2020	Kazakhstan	Plasma	68 (33)	- miR-16-5p	0.664
					- miR-210-3p	0.713
					- miR-222-3p	0.760
					- miR-29c-3p	0.739
					- miR-145-5p	0.932
					- miR-191-5p	0.904
					- miR-21	0.705
					- miR-145-5p/miR-191-5p	0.984
					- miR-145-5p/miR-21-5p	0.932
					- miR-191-5p/miR-21-5p	0.919
					- miR-145-5p/miR-191-5p/miR-21-5p	0.984
Guo et al. [78]	2018	China	Serum	60 (30)	- miR-1915-3p	0.881
					- miR-455-3p	0.778
Cuk et al. [79]	2013	Germany	Plasma	180 (60)	- miR-127-3p	0.650
					- miR-148b	0.700
					- miR-376a	0.590
					- miR-376c	0.590
					- miR-409-3p	0.620
					- miR-652	0.750
					- miR-801	0.720
					- Panel of 7 miRs above	0.810
Raheem et al. [80]	2019	Iraq	Serum	60 (30)	- miR-34a	0.669
Zhu et al. [81]	2020	China	Serum	120 (60)	- miR-1908-3p	0.838
Ahmed Mohmmed et al. [82]	2021	Egypt	Serum	80 (30)	- miR-106a	0.947
Sadeghi et al. [83]	2021	Iran	Whole Blood	130 (60)	- miR-145	0.650/0.610
					- miR-106b-5p/miR-126-3p/miR-140-3p/miR-193a-5p/miR-10b-5p	0.790/0.860
Itani et al. [84]	2021	Lebanon	Plasma	73 (32)	- miR-21	0.760
					- miR-155	0.700
					- miR-23a	0.740
					- miR-130a	0.780
					- miR-145	0.810
					- miR-425-5p	0.830
					- miR-139-5p	0.830
					- miR-451	0.730
					- miR-145/miR-425-5p	0.830
					- miR-21/miR-23a	0.800
					- miR-21/miR-130a	0.820
					- miR-21/miR-23a/miR-130a	0.820
					-miR-145/miR-139-5p/mir-130a	0.960
					- miR-145/miR-139-5p/mir-130a/miR-425-5p	0.970
Mahmoud et al. [85]	2021	Egypt	Serum	95 (25)	- miR-185-5p	0.838
					- miR-301a-3p	0.899
Zou et al. [86]	2022	Multiple	Serum	374 (197)	- miR-133a-3p/miR-497-5p/mir-24-3p/miR-125b-5p/miR-377-3p/miR-374c-5p/miR-324-5p/miR-19b-3p	0.918
				379 (199)	- miR-133a-3p/miR-497-5p/mir-24-3p/miR-125b-5p/miR-377-3p/miR-374c-5p/miR-324-5p/miR-19b-3p	0.915
				325 (199)^d^	- miR-133a-3p/miR-497-5p/mir-24-3p/miR-125b-5p/miR-377-3p/miR-374c-5p/miR-324-5p/miR-19b-3p	0.916
				210 (199)^e^	- miR-133a-3p/miR-497-5p/mir-24-3p/miR-125b-5p/miR-377-3p/miR-374c-5p/miR-324-5p/miR-19b-3p	0.953
Zou et al. [87]	2021	Singapore	Serum	369 (100 + 196)	- miR-451a/miR-195-5p/miR-126-5p/miR-423-3p/miR-192-5p/miR-17-5p	0.873
Li et al. [88]	2022	China	Serum	98 (49)	- miR-9-5p	0.852/0.937
					- miR-17-5p	0.706/0.652
					- miR-148a-3p	0.866/0.875
Shaker et al. [89]	2021	Egypt	Serum	450 (150 + 120)	- miR-29	0.916
					- miR-182	0.970
Uyisenga et al. [90]	2021	Rwanda	Plasma	45 (18)	- let-7a-5p/miR-150-5p/miR-940/miR-32-5p/miR-342-3p/miR-33a-5p/miR-130a-3p/let-7i-5p/miR-328-3p/miR-29b-3p/miR-146a-5p/miR-29a-3p/miR-126-3p	0.868
					- let-7a-5p/miR-150-5p/miR-940/miR-32-5p/miR-33a-5p/miR-130a-3p/miR-185-5p/let-7i-5p/miR-328-3p/miR-29b-3p/miR-146a-5p/miR-210-3p/miR-126-3p	0.865
					- let-7a-5p/miR-150-5p/miR-940/miR-32-5p/miR-33a-5p/miR-130a-3p/let-7i-5p/miR-328-3p/miR-29b-3p/miR-210-3p/miR-126-3p	0.865
					- let-7a-5p/miR-150-5p/miR-940/miR-32-5p/miR-342-3p/miR-33a-5p/miR-130a-3p/let-7i-5p/miR-328-3p/miR-29b-3p/miR-146a-5p/miR-210-3p/miR-126-3p	0.865
					- let-7a-5p/miR-150-5p/miR-940/miR-32-5p/miR-33a-5p/miR-130a-3p/miR-185-5p/let-7i-5p/miR-328-3p/miR-29b-3p/miR-146a-5p/miR-29a-3p/miR-126-3p	0.863
					- let-7a-5p/miR-150-5p/miR-940/miR-32-5p/miR-33a-5p/miR-130a-3p/let-7i-5p/miR-328-3p/miR-29b-3p/miR-146a-5p/miR-210-3p/miR-126-3p	0.863
					- let-7a-5p/miR-150-5p/miR-940/miR-32-5p/miR-33a-5p/miR-130a-3p/let-7i-5p/miR-29b-3p/miR-146a-5p/miR-210-3p/miR-126-3p	0.861
					- let-7a-5p/miR-150-5p/miR-940/miR-33a-5p/miR-130a-3p/miR-328-3p/miR-29a-3p/miR-126-3p	0.859
					- let-7a-5p/miR-150-5p/miR-940/miR-32-5p/miR-33a-5p/miR-130a-3p/let-7i-5p/miR-328-3p/miR-29b-3p/miR-29a-3p/miR-126-3p	0.859
					- let-7a-5p/miR-150-5p/miR-940/miR-32-5p/miR-33a-5p/let-7i-5p/miR-29b-3p/miR-146a-5p/miR-29a-3p/miR-126-3p	0.859
					- let-7a-5p/miR-150-5p/miR-940/miR-32-5p/miR-130a-3p/miR-185-5p/let-7i-5p/miR-29b-3p/miR-146a-5p/miR-126-3p	0.859
					- let-7a-5p/miR-150-5p/miR-940/miR-130a-3p/miR-328-3p/miR-29a-3p/miR-210-3p/miR-126-3p	0.857

^a^Country from which the cases and controls of the reported model were sampled

^b^Sample size (cases, controls and benign) of the reported model

^c^For each reported model, its ROC AUC is shown. If not available, then the sensitivity and specificity pair are reported

^d^Model with cases up to TNM stage II

^e^Model with TNM stage III and IV cases

The 56 studies reported a total of 173 different models. Among them, 121 analysed single miRNA performance, which covered a total of 68 unique miRNAs. On the other hand, 52 models analysed panels of miRNAs and their performance, covering 55 unique miRNAs. Moreover, 82 models had plasma as the specimen type, 81 had serum, while 10 had whole blood. It is worth restating that, in addition to the analyses performed on all the reported models, this meta-analysis also evaluates one model per study (*n* = 56), the most important model per study.

### QUADAS-2 risk of bias assessment

**Fig. 2:**
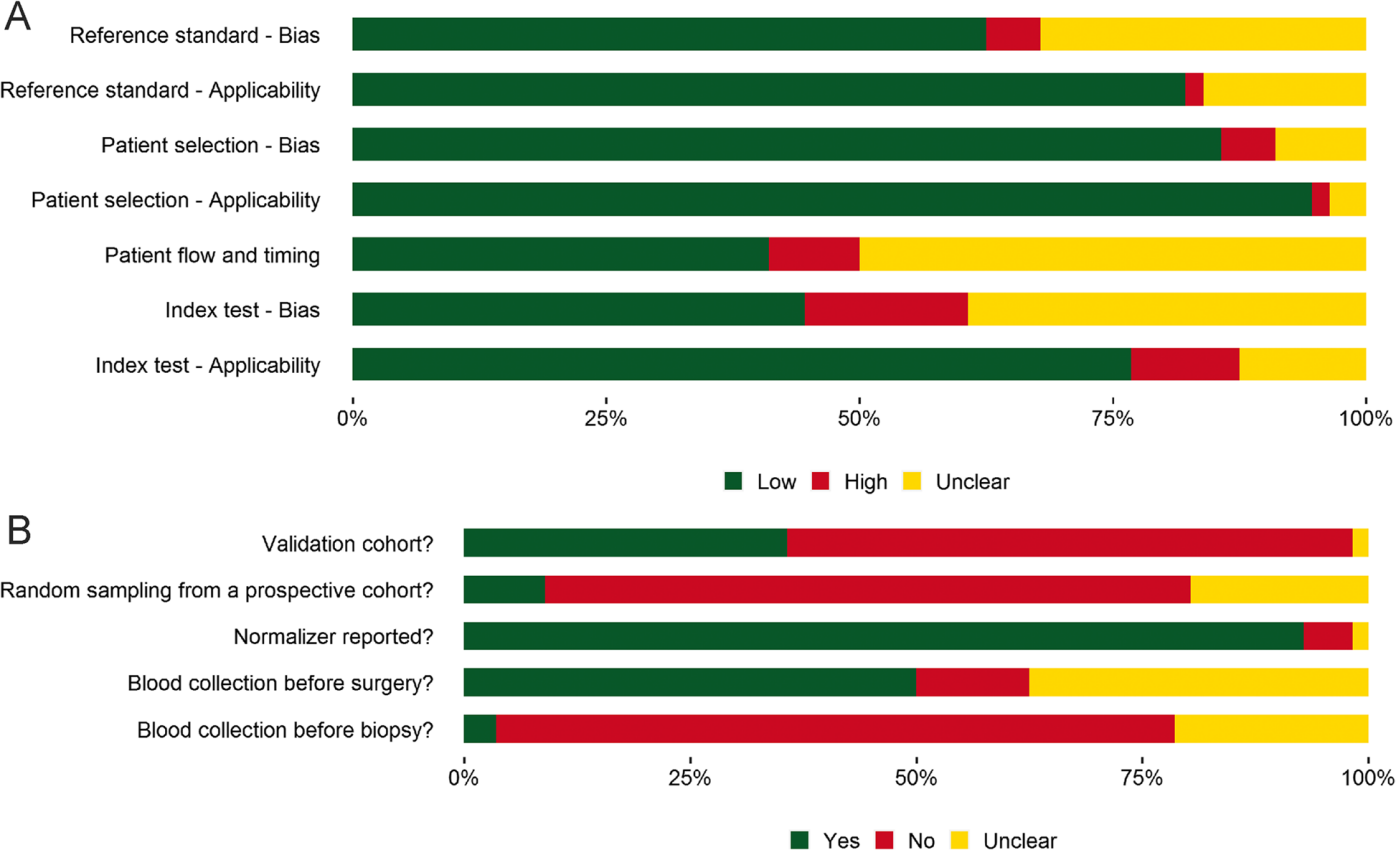
Summary of the QUADAS-2 evaluation performed on 56 articles. Proportions of Low risk of bias (Yes), Unclear and High risk of bias (No) are shown for **A**) key questions on applicability and bias and **B**) most important signalling questions

The QUADAS-2 assessment was performed on the 56 included studies. More than 75% of studies had a low probability of having an index test and patient selection applicability concern, while 82.1% of studies had low probability of having a reference standard applicability concern. On the other hand, 41.1% of the studies had low risk of bias within the patient flow and timing category. Despite the low probability of applicability concern for the index tests for the majority of the studies, only 44.6% had a low probability of risk of bias coming from the index test. Nevertheless, in the index test category, only 16.1% of the studies had a high probability of bias (Fig. 2a). Interestingly, only 8.9% of the studies performed or explicitly stated that prospective sampling, without knowing the status of the cases and controls was performed. This is also associated with the fact that in most meta-analysed studies blood was collected after the biopsy was performed on the patient. Additionally, 50% of studies explicitly stated that blood collection was performed before surgery (Fig. 2b).

### Descriptive statistics

**Fig. 3:**
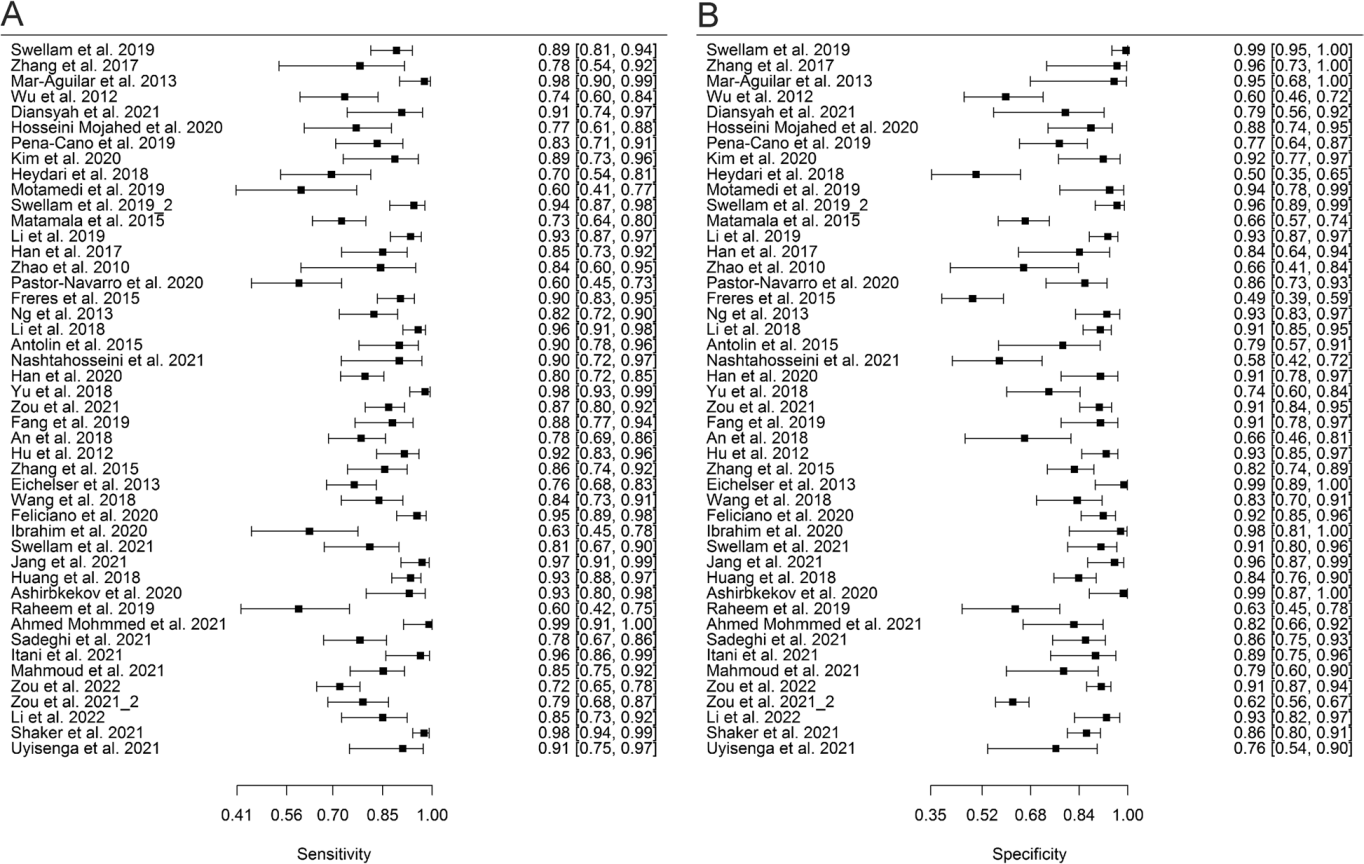
Forest plot of **A**) sensitivities and **B**) specificities of the most important model from each study. The respective values and their confidence intervals can be seen on the right side of each plot

Both sensitivity and specificity reports were heterogeneous across models (sensitivity: X^2^ = 1171.8, *p* < 0.001; specificity: X^2^ = 1019.3, *p* < 0.001). In addition, on the same group of models, a negligible positive correlation *r* = 0.09 [-0.08—0.25] of sensitivities and false positive rates (FPRs) was found. Forest plots of sensitivity and specificity were based on the most important models per study and can be seen in Fig. 3a and b, respectively.

### Bivariate analysis

**Fig. 4:**
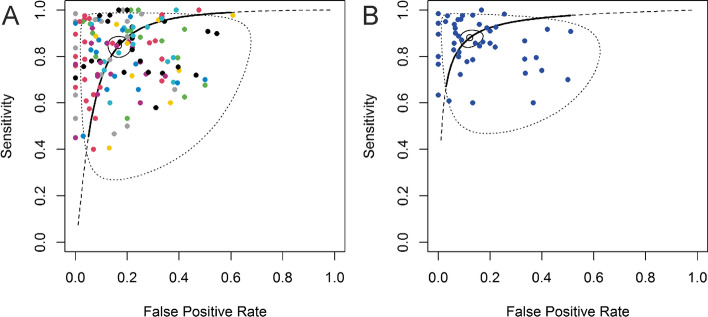
SROCs of the bivariate models. **A** SROC of all reported models. Points with the same colour in the graph represent models which come from the same study. **B** SROC of the most important model from each study

A pooled estimate of 0.85 was obtained for sensitivity and 0.83 for specificity on all the reported models with performance data (146 models). For the most important model per study (46 models), slightly better pooled sensitivity (0.88) and specificity (0.88) were obtained. Confidence intervals as well as the variances of logit transformed sensitivity and FPR and correlation estimates for both bivariate models can be found in Table 3. The summary receiver operating characteristic curves (SROCs) of the two models are shown in Fig. 4a and b.

**Table 3:** Summary of the bivariate analyses on all reported models and on most important model per study

	**Fixed Effects**			**Random Effects**					
				**Model**			**Study**		
		**Estimates**	**CI**	**Std.Dev**	**Corr**	**n**	**Std.Dev**	**Corr**	**n**
**All reported models**	**Sensitivity**	0.85	[0.81—0.88]	0.85	-0.17	146	0.70	0.06	46
	**Specificity**	0.83	[0.79—0.87]	0.60	-0.17	146	0.74	0.06	46
**Most important models**	**Sensitivity**	0.88	[0.85—0.91]	0.86	0.23	46			
	**Specificity**	0.88	[0.84—0.91]	1.00	0.23	46

To take into account the experimental and study-design differences among studies, fixed effects were added to the bivariate mixed models (specimen type, normalizer, single or multiple miRNA panel and inclusion of stage III and/or stage IV cases). The significant fixed effects for all models were the single or multiple panel type as well as the normalizer type, whereas for the most important models there were no significant fixed effects. Details on the fixed effect models can be found in Supplementary Tables S2 and S3 (Additional file 2).

### Influence analysis and outliers

Outlier analysis was performed on the complete set of models and was based on the odds ratio. Models with an odds ratio of 2 standard deviations (SDs) away from the mean were considered outliers. A total of 5 models were identified as outliers.

**Fig. 5:**
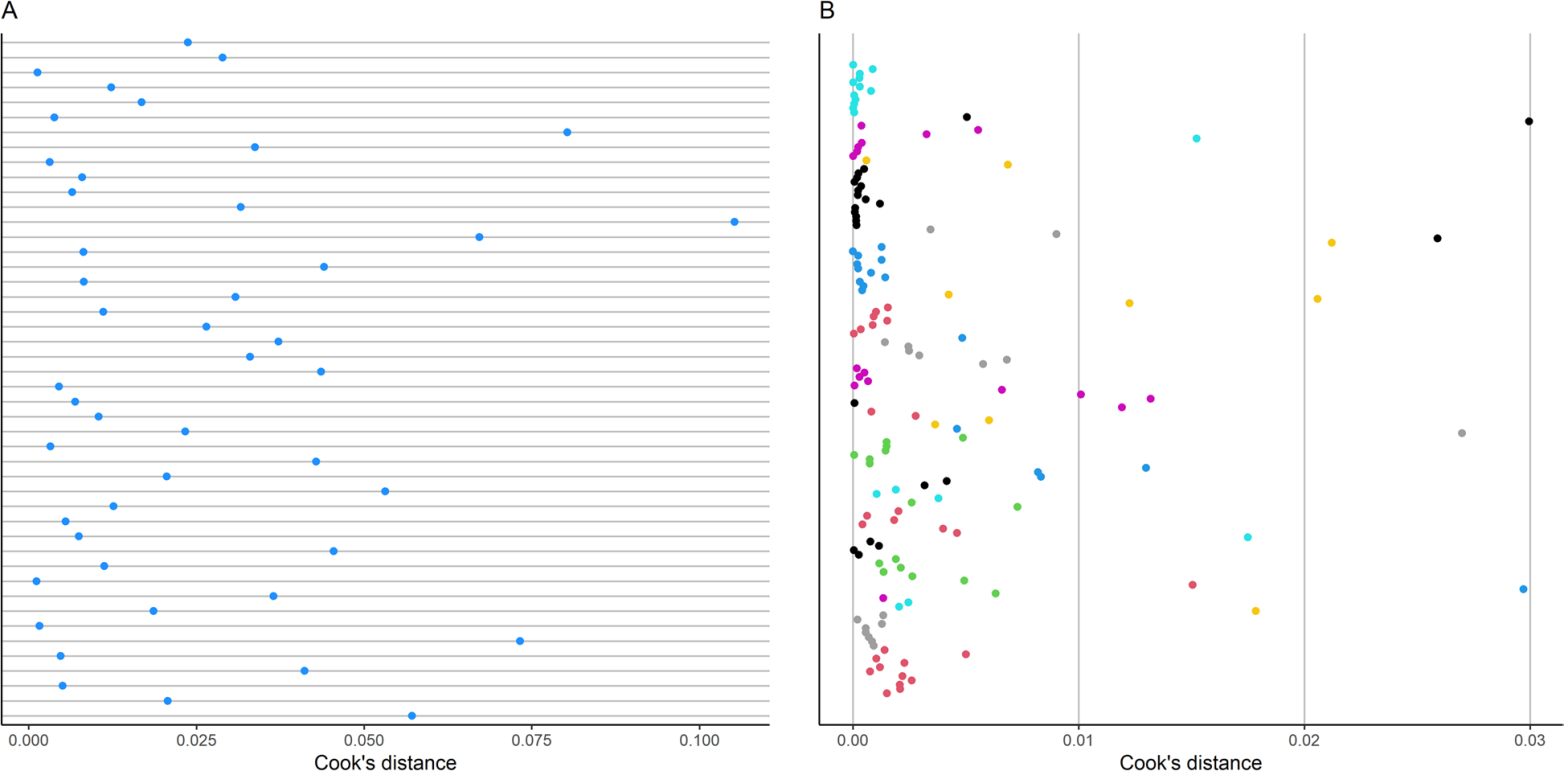
The calculated influence analysis was represented in Cook's distance units. **A** Influence analysis of most important models from each study. **B** Influence analysis of all reported models where the points with the same colour represent models which come from the same study

In order to detect influential models in the two generalized linear multilevel models mentioned above, Cook’s distances of the included models were calculated (Fig. 5a and b). Models with a Cook’s distance more than 2 SDs away from the mean were deemed as very influential. On all reported models, 8 of them were influential. Interestingly, none of the models from the outlier analysis matched the ones obtained from the influence analysis. Generalized linear multilevel models without the influential models were fit in order to determine statistical robustness; a pooled estimate of 0.84 [0.80—0.87] was obtained for sensitivity and 0.84 [0.80—0.88] for specificity. On the most important model per study, 3 models were found to be influential. After repeating the generalized linear multilevel model, pooled sensitivity and specificity were 0.87 [0.84—0.90] and 0.86 [0.82—0.89], respectively. A very modest discrepancy is observed between the bivariate analyses with and without the influential models. This was observed for estimates on both all and most important models, indicating the robustness of the pooled estimates.

### Publication bias

**Fig. 6:**
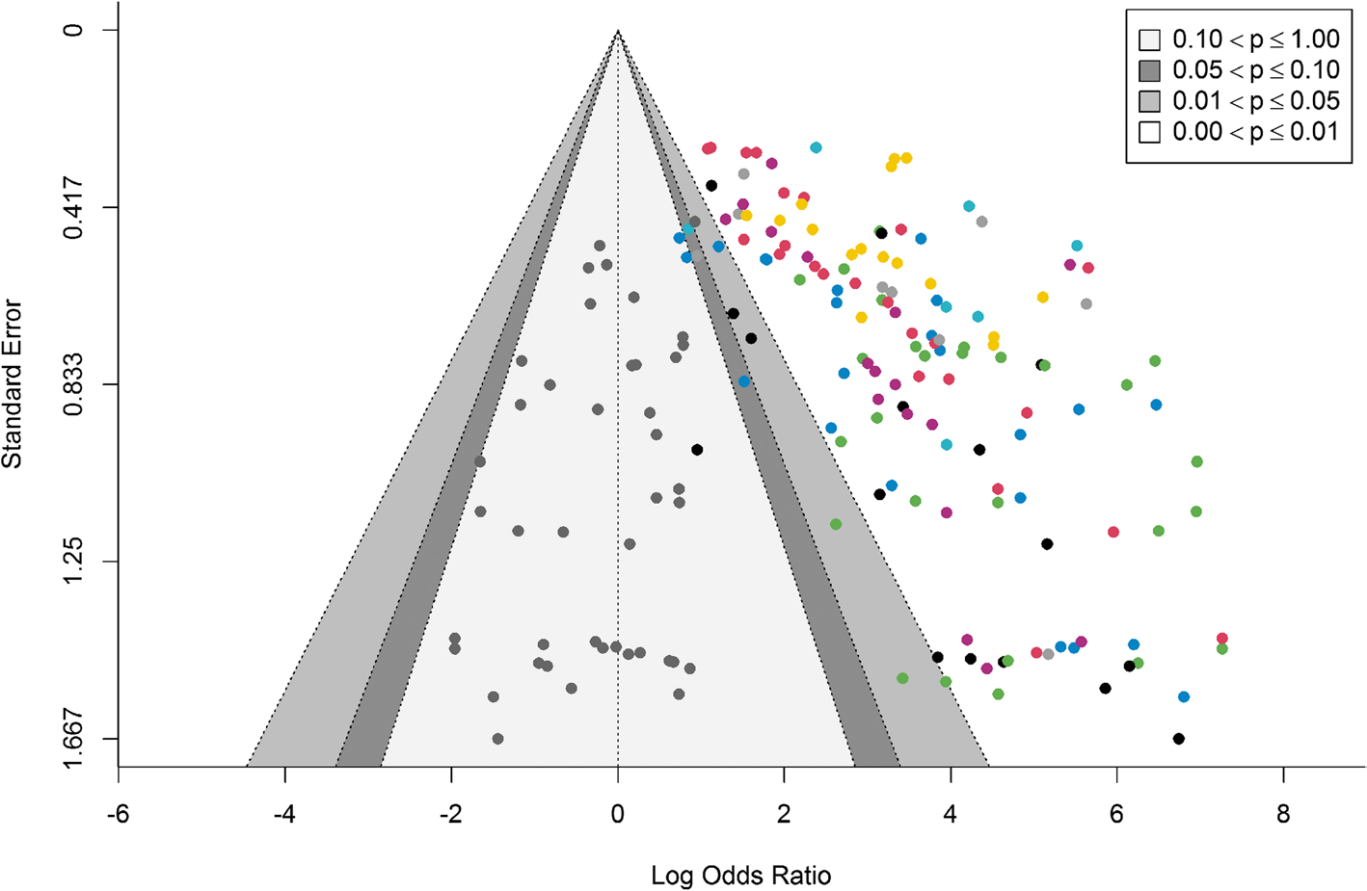
Publication bias was performed on all reported models. Points with the same colour in the graph represent models which come from the same study. The cluster of grey points on the left-hand side of the graph represents the missing models which would be required in order not to have a publication bias

Publication bias was evaluated for all the reported models. A funnel plot was generated on the log odds ratio and standard error (Fig. 6). Egger’s test, in which a random effect on the studies was added, was used to test for publication bias. A *p*-value of < 0.001 indicated a potential publication bias.

### Subgroup bivariate analysis

In order to determine performance differences between methodological variations in the studies as well as to evaluate some potential candidate sources of between-study heterogeneity, subgroup analyses were performed. The main subgroups considered were: single vs multiple (panel) miRNAs, plasma vs serum specimen type, studies including stage III and/or IV BC cases vs studies not including stage III and/or IV BC cases, exogenous vs endogenous normalizer and stratification of studies by QUADAS-2 performance. The subgroup analyses based on all reported models were performed utilizing generalized linear multilevel models with both random effects on study and models.

**Fig. 7:**
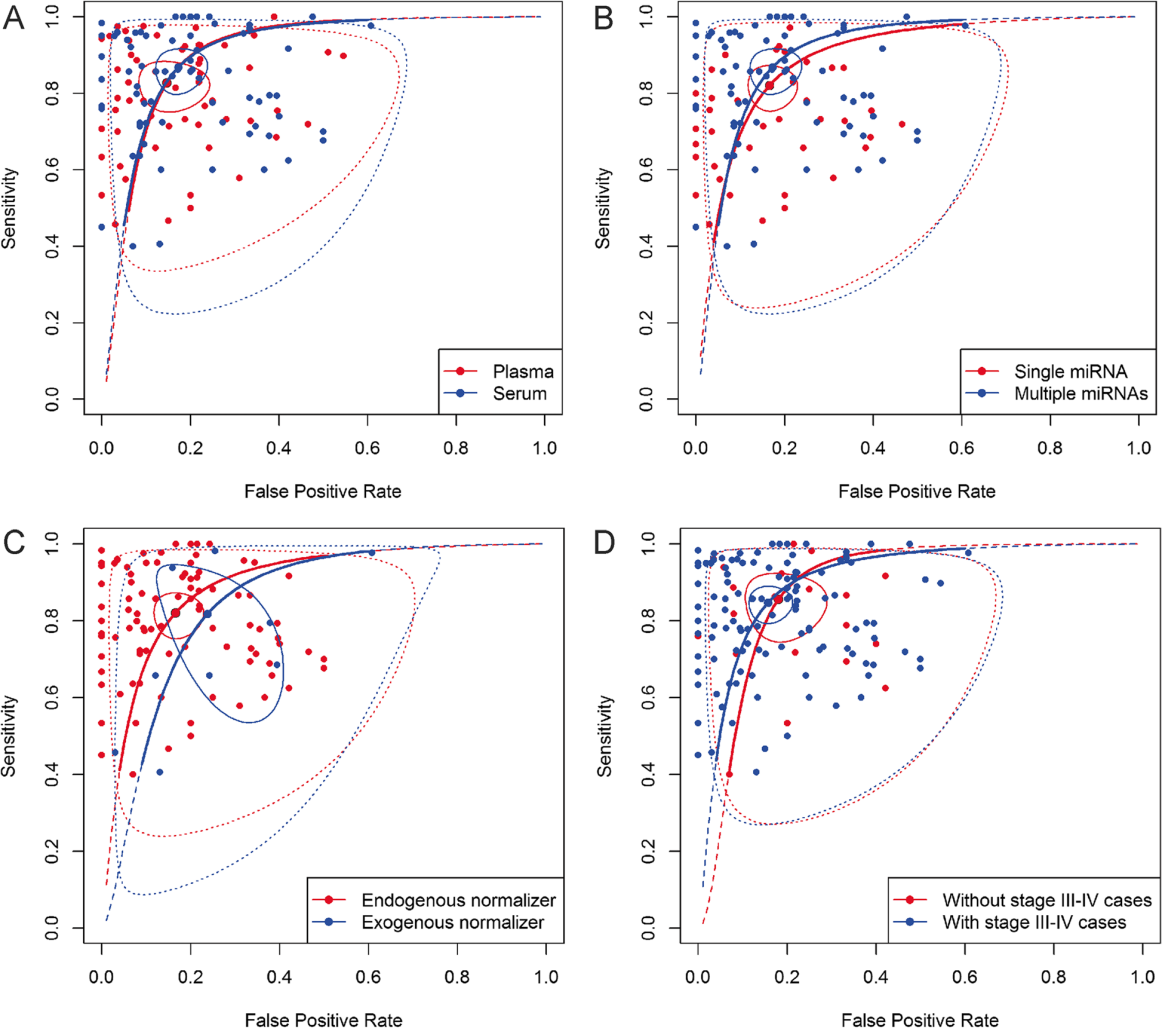
SROCs of the subgroup bivariate models based on all reported models. **A**) Plasma vs Serum **B**) Single vs Multiple panel miRNAs **C**) Endogenous v Exogenous normalizer **D**) With vs Without stage III and stage IV cases

Pooled sensitivity and specificity on plasma models were 0.83 [0.77—0.87] and 0.85 [0.78—0.91], respectively, while for serum the pooled sensitivity and specificity were 0.87 [0.81—0.91] and 0.83 [0.78—0.87], respectively (Fig. 7A). On average, models based on miRNA panels perform better than models based on a single miRNA. The former subgroup had a pooled sensitivity and specificity of 0.90 [0.86—0.93] and 0.86 [0.80—0.90], respectively, while the latter subgroup had a pooled sensitivity and specificity of 0.82 [0.77—0.86] and 0.83 [0.78—0.87], respectively (Fig. 7B). Considering the sample size disparity between models that used exogenous and endogenous normalizers, the performance between the two groups is quite similar, with the endogenous based models having a higher specificity (Fig. 7C). For models with an exogenous normalizer, the pooled sensitivity and specificity were 0.82 [0.60—0.93] and 0.76 [0.63—0.86], respectively, while the pooled sensitivity and specificity for models with an endogenous normalizer were 0.82 [0.77—0.86] and 0.83 [0.78—0.87], respectively. Expectedly, models without stage IV BC samples and models with < 4.5% stage IV BC samples performed similarly when the pooled sensitivities and specificities were compared. The models without stage IV cases had a pooled sensitivity of 0.85 [0.81—0.88] and specificity of 0.84 [0.80—0.88], while models with stage IV cases had a slightly better pooled estimate where the sensitivity was 0.87 [0.61—0.97] and specificity was 0.86 [0.80—0.90]. This slight difference could be attributed to the difference in model numbers analysed in the two groups, as can be seen from the confidence interval for the sensitivity estimate for models with stage IV cases. Thus, since low between-study heterogeneity was observed in this subgroup analysis, the total cohort of models which includes both with (< 4.5%) and without stage IV BC samples can be considered reliable for assessing general ability of circulating miRNAs to diagnose BC, with the condition that the models assessed do not have a higher percentage of stage IV cases than would be observed in community screening for BC. To further investigate the impact of stages on diagnostic performance, a subgroup analysis of the models with and without stage III and IV was performed. Pooled sensitivity and specificity of 0.84 [0.80—0.88] and 0.85 [0.80—0.88], respectively, were obtained for the former group, while of 0.86 [0.77—0.91] and 0.82 [0.74—0.88], respectively, for the latter (Fig. 7D). As observed in the previous subgroup analyses, models which include later BC stages (III and IV) have a slightly better diagnostic performance when compared to models which include only earlier stages (0, I and II). SROCs of the subgroup analyses on the most important model of each study can be found in Supplementary Fig. 1 (Additional file 3). Interestingly, when studies were stratified based on the QUADAS-2 performance cut-points (no cut-point, > 3, > 4 and > 5 “low” on the seven key questions), increasing QUADAS-2 score corresponded to decreasing pooled diagnostic performance, chiefly reflected in specificity. This was observed on all reported models as well as on the most important model per study. Details on results of subgroup analysis on all reported models and on the most important model per study can be found in Supplementary Table S4 and S5, respectively (Additional file 2).

Lastly, we estimated the pooled sensitivity and specificity on all reported models for each year to assess if there is a diagnostic performance trend throughout the years. A linear regression was performed on pooled sensitivities and specificities and no significant linear association was found (Supplementary Fig. 3—Additional file 3).

### miRNA-21-5p

miRNA-21-5p is the most commonly analysed miRNA among the included studies in this meta-analysis. Therefore, we performed a bivariate analysis using the generalized linear multilevel model in order to meta-analyse the diagnostic ability of circulating cell-free miRNA-21-5p in BC. The pooled sensitivity and specificity for models evaluating only miRNA-21-5p were 0.74 [0.64—0.83] and 0.81 [0.70—0.89], respectively. The SROC and the details on the model can be seen in the Supplementary Fig. 2 (see Additional file 3) and Supplementary Tables S4 and S5 (Additional file 2).

### Univariate analysis on log-DOR

In order to include studies not reporting diagnostic accuracy in terms of sensitivity and specificity we performed a univariate analysis on log-DOR using the q-Point data from the reported ROC graphs. The q-Point was extracted for all models with a ROC curve. A pooled log-DOR based on all reported models of 2.48 [2.15 – 2.81] resulted. Significant heterogeneity was observed in the model (Cochran’s Q = 978.9, *p* < 0.001). As there was a large difference in the number of models that used endogenous and exogenous normalizers, we complemented the bivariate subgroup analysis on endogenous versus exogenous models with the log-DOR univariate analysis where the difference in the model numbers is smaller. The estimate of pooled log-DOR for endogenous models is 2.58 [2.22—2.94], while for the exogenous models it is 1.45 [0.86 – 2.04], confirming the discrepancy in diagnostic accuracy found with bivariate models. The log-DOR estimate details of the mentioned models as well as all the other models are found in Supplementary Table S6 and S7 (Additional file 2).

### Preference for sensitivity or specificity

**Fig. 8:**
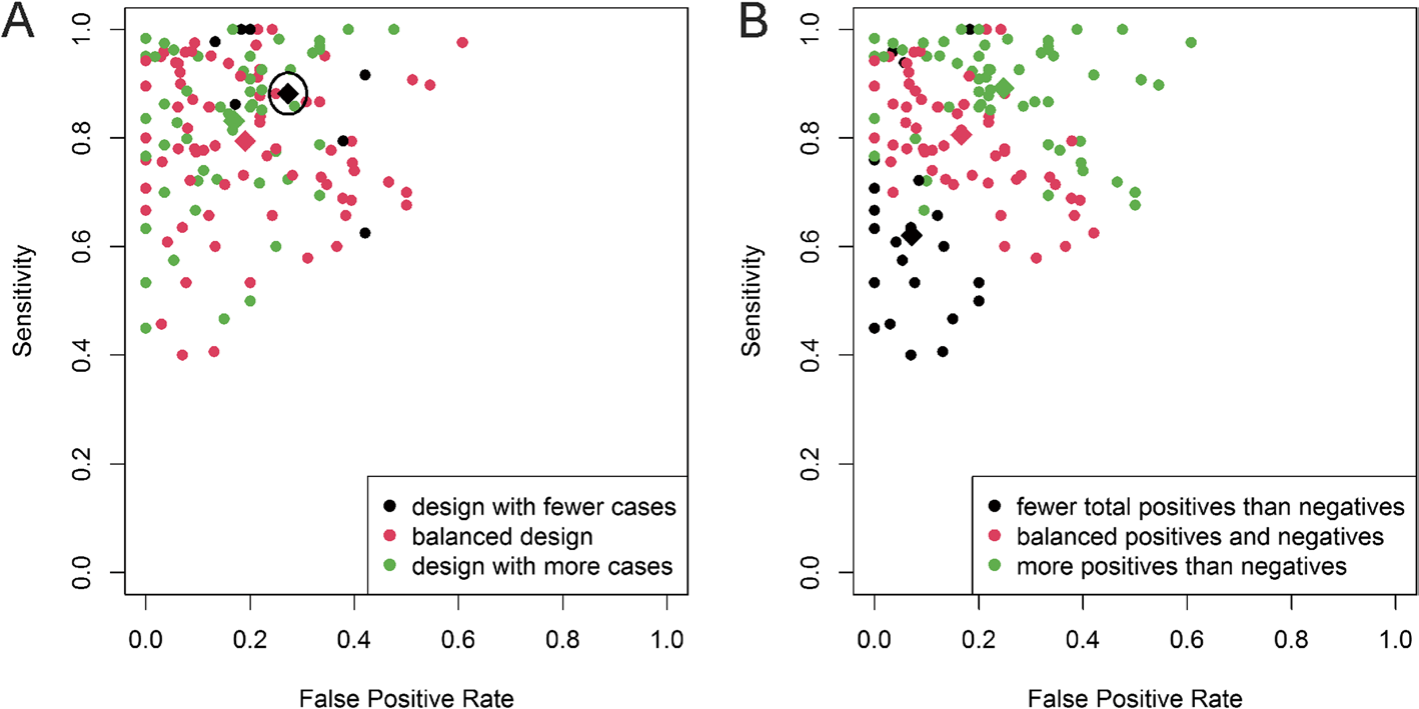
Comparison of diagnostic performance of models to their imbalance of proportions of **A**) cases to controls or **B**) predicted positive to predicted negative screens, represented by a colour which corresponds to one of the three imbalance of proportions cut-point groups. Diagnostic performance means (with the confidence intervals) of the three ratio groups are represented by diamonds

To investigate whether a preference of a model for sensitivity/specificity is related to an imbalance of proportions between cases and controls or to predicted positive (TP + FP) and predicted negative (TN + FN) samples, a graphical technique was employed: models were divided in three groups according to the proportion of cases to controls or predicted positive to predicted negative samples, coloured and plotted on a ROC plane (Fig. 8).

Differences in model designs based on the proportion of cases to controls are mainly reflected in the FPR (Fig. 8a), as models with fewer cases than controls tend to have a larger FPR. Overall, models with a balanced case–control design or a design with more cases than controls are far more abundant than models with fewer cases than controls. A clearer performance trend can be seen when the proportion of the positive screens and negative screens is taken into account (Fig. 8b). Models with fewer positive screens than negative usually tend to have a smaller FPR and sensitivity, while models with more positive screens than negative have the tendency for the opposite performance characteristics, with larger FPR and sensitivity. Those models with balanced positive and negative screens have more balanced FPR and sensitivity when compared to the previous two groups. In sum, sample composition, i.e. ratio of cases to controls, seems to influence diagnostic accuracies, probably via study level model tuning. Moreover, the predicted positive and predicted negative ratio is most likely influenced by the compromise or preference between sensitivity and specificity.

#### Quantifying the author or model preference for sensitivity or specificity

**Fig. 9:**
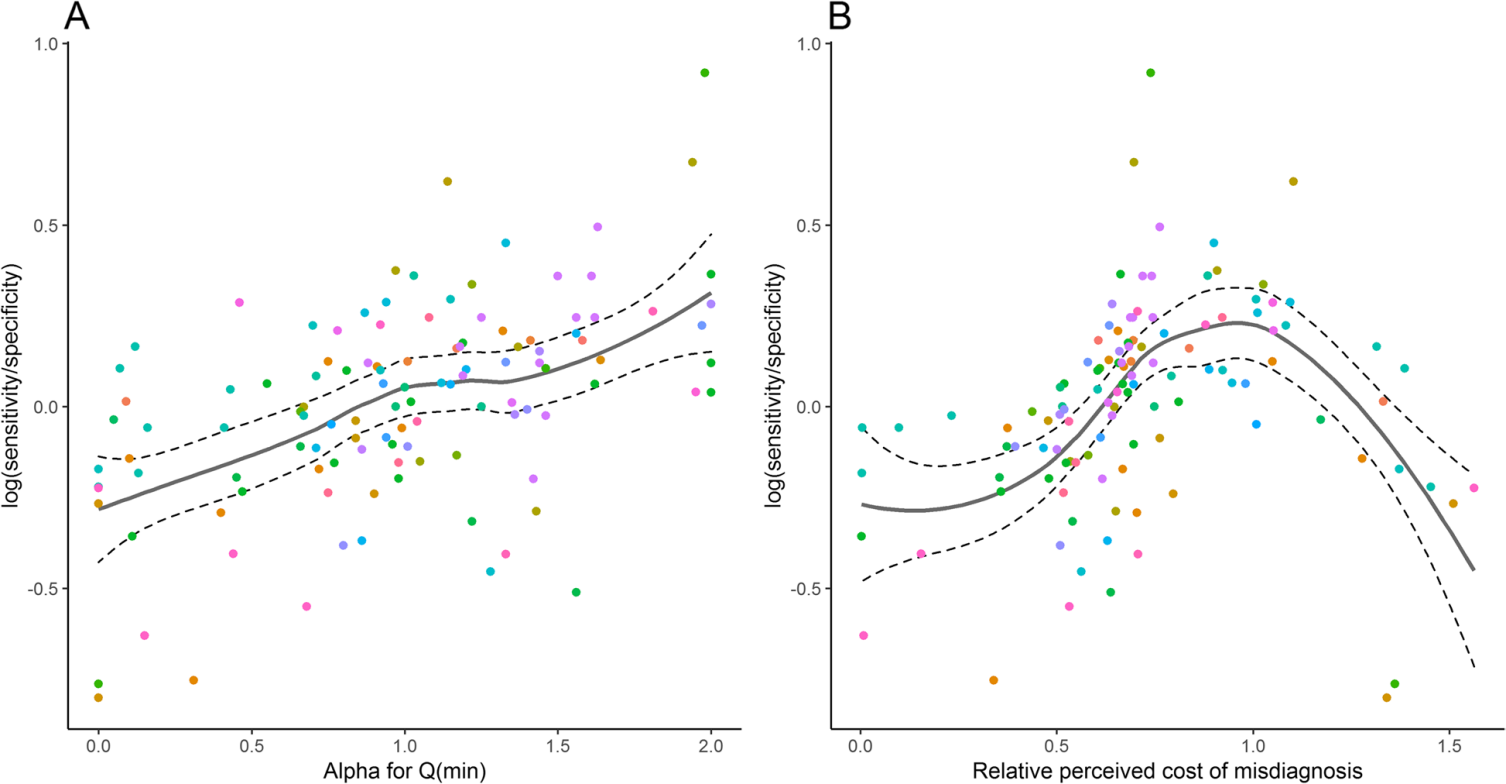
Preference estimates based on log (sensitivity/specificity) for all reported models using **A**) alpha for minimum Q and **B**) relative perceived cost of misdiagnosis (c_1_). Points with the same colour in the graph represent models which come from the same study

By utilizing the alpha parameter, we assessed from the ROC shape if the meta-analysed models preferred sensitivity or specificity (Fig. 9a). A general trend of preference can be seen in the plot. However, since the trend is not strong enough, only the models with an alpha z-score > 0.8 SDs away from the mean were considered as studies with some kind of preference. Based on the mentioned alpha parameter, 25 of the 117 analysed models had a preference for sensitivity, while 24 had a preference for specificity. The preference is derived from ROC curve shape, so a preference in shape does not necessarily imply that the pair of sensitivity and specificity at the authors’ preferred cut-off value reflects this preference: 22 out of the 25 models considered to prefer sensitivity had a higher sensitivity than specificity, while 18 out of the 24 models considered to prefer specificity had a higher specificity.

In addition to the assessment of preference of the model by the alpha parameter, we assumed that in all the models the study authors base their decision about the cut-off value on a perceived cost c_1_ for not detecting a BC patient and a cost c_0_ for a positive screen on a healthy person. Recall that the perceived cost c_1_ is calculated in units of c_0_ = 1 (Fig. 9b) and note that the prevalence factor was omitted. The strength of the preference trend is similar to that of the previous plot. Hence, models with a c_1_ z-score of > 0.8 SDs away from the mean were considered as studies with some kind of author preference. Based on the c_1_ value, 10 of the 117 analysed models had a preference for sensitivity, while 80 had a preference for specificity. From the 80 models which were considered to prefer specificity 41 had a higher specificity than sensitivity. Interestingly, most of the models with a high c_1_ value (> 0.8 SD) did not have a higher sensitivity compared to specificity, a consequence of the underlying ROC curve shapes. In this sense, most of the ten models did not have a preference for sensitivity in the naive sense. Until the c_1_ starts surpassing the value of 1, the plot seems to be linear and in concordance with the plot in Fig. 9a. Hence, the alpha parameter preference method has shown more robust results. Having said that, it is worth noting that between the two preference assessment methods, there were 12 common models which preferred specificity and 23 common models which did not have a significant preference. No common models were found for sensitivity preference.

## Discussion

As circulating cell-free miRNAs are promising biomarkers for the (early) detection of BC and as there have been numerous diagnostic circulating miRNA studies in the recent years [6], in this study we have attempted to evaluate the overall diagnostic performance capability of the thus-far reported circulating miRNA-based screenings. In addition, one important segment which we touched upon is the lack of standardization between the studies as well as other factors which might be the cause of some discordant results and of a lack of commonly appearing miRNAs which could be clinically viable diagnostic biomarkers. The pooled sensitivity (0.85) and specificity (0.83) obtained on all the reported models was quite satisfactory, especially considering the fact that even the models which did not perform as well were included in the pool. The obtained estimate of the pooled sensitivity is quite robust and reliable: after repeating the bivariate analysis without the influential models a very similar pooled sensitivity (0.84) and specificity (0.84) were obtained. It is important to note, however, that a highly significant publication bias was observed based on the Egger’s test, which could also hint at a tendency of primary report authors to report top performing models instead of all a priori plausible models. In addition, studies tend to have slightly worse diagnostic performance, mainly reflected in specificity, when having a lower probability of bias or lower probability of poor applicability. Moreover, single or multiple miRNA panel and normalizer type were significant fixed effects in the bivariate model on all reported models. The significance of the fixed effects is also confirmed by the subgroup analyses as we see a significantly better performance, especially in sensitivity, of multiple miRNA panels compared to single, as well as a better pooled performance of models utilizing endogenous compared to exogenous normalizers. Considering that in the bivariate analysis there was a sample disparity between the models which used endogenous and exogenous normalizers, the issue was less severe in the univariate analysis based on the log-DOR. Nevertheless, in the univariate analysis we also observed that models based on endogenous normalizers perform better than exogenous normalizers.

Multiple different endogenous and exogenous normalizing miRNAs/genes have been used both in the meta-analysed studies and in studies working with circulating miRNAs in other fields. However, none of them were found to be an optimal solution for normalizing qRT-PCR miRNA data [91]. Hence, normalizer is one of the most important factors which contributes to the heterogeneity of results. One solution to the normalizer issue which might produce more consistent results, as proposed by [91], is to use ratio-based normalization where the ratio of two miRNAs is compared between cases and controls. Only one study [64] out of the 56 which we meta-analysed used the ratio-based normalization. Mimics of miRNAs and mean threshold cycle of 50 miRNAs with the highest mean expression were two other types of normalization methods found within three distinct meta-analysed studies [53, 78, 90]. However, we believe that the lack of experimental practicality and efficiency of the former and the lack of between-study comparability of the latter method may limit the use of such normalization methods in a standardized way. Although not significant in the fixed effect model, a slight diagnostic performance difference between models with and without stages III and IV was observed. The same is true for models with and without stage IV. This indicates that the stage distribution could play a role in the between-study bias. Two other important factors could contribute to the increase of more consistent results: the usage of validation cohorts and random selection of cases and controls with prospective sampling [92]. As can be seen in Fig. 2b, only about 40% of the studies used a validation cohort while no study performed or explicitly stated that they performed prospective sampling without knowing the status of cases and controls. Independent internal/external cohorts are a fundamental requirement in the process of biomarker validation, while prospective random sampling would enable a non-biased and generalizable biomarker evaluation [92] as well as sampling of blood before biopsy. Blood sampling before biopsy would allow to minimize the influence of biopsychological/physical effects that could also influence the level of circulating miRNAs [93]. Despite not significant in this meta-analysis, differences in specimen type might influence the heterogeneity of the obtained results. Utilizing plasma as specimen type runs the risk of having hemolysed samples which affects the miRNA content of the samples [94–96] as plasma contains cellular components that may contribute miRNAs from apoptotic or lysed cells (e.g., red blood cells, platelets). Therefore, studies using plasma as the specimen type need to check for hemolysed samples and exclude them [97] or to evaluate the influence of potential hemolysis on candidate miRNAs before their analysis in plasma samples [98]. On the other hand, during coagulation of serum samples, RNA molecules are released and may change the true profile of circulating miRNAs [96]. Hence, these issues are of crucial importance in order to standardize the procedure of circulating miRNA detection. Taken together, in order to obtain clinically viable diagnostic miRNAs which could be applied on the target population (women eligible for routine mammographic screening), a standardized laboratory protocol should be created. Additionally, future studies with random case–control selection from prospective sampling of women undergoing routine screening will allow for a standardized stage distribution and a higher applicability of novel diagnostic biomarkers to the target population.

Among the meta-analysed models, there were slightly more models with a balanced case–control ratio than models with significantly more cases than controls. Models with significantly less cases were less common than the previous two groups. Sensitivity across the three groups seemed to be consistent, while the group with significantly less cases tends to have a larger FPR. Thus, the ratio of cases and controls has an effect on diagnostic accuracies while the ratio of predicted positive and predicted negative screens is influenced by or is a resemblance of the model’s preference for sensitivity or specificity.

Either due to the model designs or authors’ perceived costs of misdiagnosis, for some models a slight preference for sensitivity or specificity was observed. Such a trend could clearly be obtained with the alpha method as many of the studies which were predicted to prefer sensitivity or specificity actually had the higher respective diagnostic performance value. On the other hand, the method based on the authors’ perceived cost of misdiagnosis is not as robust as it yielded a much larger number of models which prefer specificity to sensitivity than models which prefer sensitivity to specificity, where many of the models in the groups did not have a higher respective diagnostic performance statistic.

Two meta-analyses on BC diagnostic circulating miRNAs were performed in 2014 [20, 21]. Seventeen unique studies were meta-analysed in the two studies. Seven out of the 17 studies were included in this meta-analysis. One of the main differences in exclusion criteria between our study and the mentioned two studies is the fact that we excluded studies with > 4.5% stage IV cases. The reason for this being that we expected an overestimation in diagnostic performance in studies which include a larger percentage of stage IV cases than would be expected in BC community screens [99]. The pooled sensitivity and specificity obtained in this study is in concordance with [20]. However, [21] have obtained a slightly lower pooled sensitivity and slightly higher specificity. This suggests that the overall diagnostic performance of circulating miRNAs on detection of BC has not significantly improved over the years. On the other hand, the pooled diagnostic performance obtained from the most important model of each study has shown an improvement in both sensitivity and specificity. Interestingly, the percentage of studies with high, low and unclear evaluations on the four key domains of QUADAS-2 were very similar between this study and [21]. As it is the most commonly analysed miRNA among the meta-analysed studies, we have evaluated the pooled sensitivity and specificity on miRNA-21-5p. A study in 2014 [100] performed a meta-analysis on BC diagnostic serum miRNA-21. Marginally lower pooled sensitivity and specificity on miRNA-21 were obtained in this study in comparison to the estimates of [100].

The main strengths of this meta-analysis are the evaluation of all the reported models from each study (as opposed to singling out one model per study), exploration of the model or author preference for sensitivity or specificity and robust, comprehensive results obtained from bivariate analyses complemented by univariate analyses when necessary. The main limitation is uncertainty due to unmodeled factors: laboratory and experimental differences, differences in stage composition of analysed cases within the studies, as well as different levels of statistical robustness of the models reported in primary studies. Another limitation is the relatively low number of databases assessed. Although we cannot exclude the possibility that we have missed some studies in the search phase, based on suggestions from the current literature with respect to database choice [101, 102] we deem the potential for systematic bias to be low. Due to their complementarity, the databases chosen for this study have around 90% median recall rate when compared to the most elaborate approach with four databases (EMBASE, MedLine, Google Scholar and Web of Science) [103].

## Conclusion

By presenting reliable estimates of diagnostic performance across studies, we have shown that diagnostic cell-free circulating miRNAs are promising biomarkers for (early) detection of BC. The subgroup analysis has revealed that multiple miRNA panels have a better pooled diagnostic performance when compared to single miRNA panels. Using novel methods to evaluate model/author preference for sensitivity or specificity, we have determined that overall, there is a tendency of the meta-analysed studies to prefer specificity. Additionally, case–control ratio likely has an impact on diagnostic accuracy, while the preference for sensitivity or specificity has an influence on the ratio of predicted positive to predicted negative screens. Prospective random sampling of cases and controls, independent validation cohorts as well as standardization of studies, especially on normalizing method, patient flow and specimen type, are of crucial importance to obtain consistent and homogenous results between studies. This would reveal reliable candidate BC diagnostic miRNA models that should be independently validated across multiple laboratories.

## Supplementary Information

**Additional file 1.** Implicit cost of misdiagnosis derivation.

**Additional file 2:** **Table S1.** Complete list of exclusion reasons and their frequencies. **Table S2.** Bivariate generalized linear mixed effect model on all reported models adjusted for covariates. **Table S3.** Bivariate generalized linear mixed effect model on most important model of each study adjusted for covariates. **Table S4**. Summary of the bivariate analysis on the all the reported models and its corresponding subgroup analyses. Subgroups marked with an asterix (*) do not have a large enough model sample size in order for the result to be reliable. **Table S5.** Summary of the bivariate analysis on the most important model of each study and its corresponding subgroup analyses. Subgroups marked with an asterix (*) do not have a large enough model sample size in order for the result to be reliable. **Table S6.** Summary of the univariate (log-DOR) analysis on all the reported models and its corresponding subgroup analysis. **Table S7.** Summary of the univariate analysis (log-DOR) on the most important model of each study and its corresponding subgroup analysis.

**Additional file 3:** **Supplementary Figure 1.** SROCs of the subgroup bivariate models based on the most important model of each study. A) Plasma vs Serum B) Single vs Multiple panel miRNAs C) Endogenous v Exogenous normalizer D) With vs Without stage III and stage IV cases. **Supplementary Figure 2.** SROCs on miRNA-21-5p bivariate models. A) miRNA-21-5p SROC of all reported models. Points with the same colour in the graph represent models which come from the same study. B) miRNA-21-5p SROC of the most important model from each study. **Supplementary Figure 3.** Pooled estimates of sensitivity and specificity calculated on all models of studies stratified by year of publication. Linear regression was performed on both sensitivity and specificity across the years and no significant linear trend was observed. For both sensitivity and specificity, the linear regression estimates were around 0.

## Data Availability

The R script as well as the dataset used for the meta-analysis is available in the GitHub repository (https://github.com/saraurru/Meta-analysis-of-diagnostic-cell-free-circulating-miRNAs-for-BC-detection).

## Abbreviations

BC: Breast cancer
MRI: Magnetic Resonance Imaging
SNP: Single Nucleotide Polymorphism
miRNA: MicroRNA
qRT-PCR: Quantitative Reverse Transcription Polymerase Chain Reaction
PRISMA-DTA: Preferred Reporting Items for Systematic Reviews and Meta-Analyses of Diagnostic Test Accuracy
NCBI PMC: PubMed Central
ROC AUC: Area Under the Curve of the Receiver Operating Characteristic
TP: True Positive
FP: False Positive
FN: False Negative
TN: True Negative
QUADAS-2: Revised tool for Quality Assessment of Diagnostic Accuracy Studies
DOR: Diagnostic odds ratio
PLR: Positive likelihood ratio
NLR: Negative likelihood ratio
PPV: Positive predictive value
NPV: Negative predictive value
SROC: Summary Receiver Operating Characteristic
SD: Standard Deviation
FPR: False Positive Rate
