# Supplementary material for: Meta-analysis of diagnostic cell-free circulating microRNAs for breast cancer detection
Source: BMC Cancer. 2022 Jun 9;22:634. doi: 10.1186/s12885-022-09698-8 (PMC9178880; doi:10.1186/s12885-022-09698-8)
Supplement: Supplementary file 1 — Additional file 1. Implicit cost of misdiagnosis derivation. [file 12885_2022_9698_MOESM1_ESM.docx]

**Implicit cost of misdiagnosis derivation**

To assess the preference, the shape of the ROC curve was analysed, adapting a parametric method of Doebler and Holling (2015) was applied. Assuming that for every study the following relationship hold:

$$t_{\alpha}\left( sens \right)=t_{\alpha}\left( spec \right)+\theta$$

where

$$t_{\alpha}\left( x \right)=\alpha\log\left( x \right)-\left( 2-\alpha\right)\log\left( 1-x \right),$$

$$x\in\left( 0,1 \right), \alpha\in(0,2)$$

so that α is a shape parameter and θ is an accuracy parameter (Doebler & Holling, 2015).

For a constant accuracy, the parameter α governs the asymmetry of the ROC curve. Hence, low values of α lead to a preference of specificity while high values lead to a preference of sensitivity. We have used estimates of α to evaluate if an individual model has inherent preference for sensitivity or specificity as well the general preference characteristics of meta-analysed models.

The tα transformation was chosen because it has been shown to be more suitable than the logit transformation (Holling et al., 2012a). Based on the extracted three points from the ROC curve, three pairs of sensitivity and specificity values, and for a set of α values, tα and θ were calculated for each point. By minimizing the heterogeneity statistic Q, estimates of α result for each model in each study (cf. Eq. 23 in Doebler & Holling, 2015).

We assume that authors base their decision about the study-level cut-off on study-specific (perceived) costs c1 for not detecting a BC patient and c1 for a positive screen of a healthy person. The cost c1 is represented in units of c0, and by setting c0=1, we simplified the calculation to this one parameter Similar to the α parameter, the c1 cost was used to evaluate if an individual model was affected by an inherent author preference as well as to evaluate the general author preference among the meta-analysed models.

For a prevalence π the expected cost is then

$$\mathbb{E}\left( cost \right)=c_{1}\pi\left( 1-p \right)+\left( 1-\pi\right)q$$

where p and q are short for sensitivity and false positive rate. Without loss of generality, the ROC curve is parametrized in q, so that p is a function of q. We can differentiate by q to obtain

$$\frac{\partial}{\partial q}\mathbb{E}\left( cost \right)=-c_{1}\pi\frac{\partial}{\partial q}p\left( q \right)+\left( 1-\pi\right)\frac{\partial}{\partial q}q= -c_{1}\pi\frac{\partial}{\partial q}p\left( q \right)+\left( 1-\pi\right)$$

The minimum cost is found at the q with

$$\frac{\partial}{\partial q}\mathbb{E}\left( cost \right) = 0$$

and we obtain:

$$c_{1}=\frac{1-\pi}{\pi} \frac{1}{p^{'}\left( q \right)}=\frac{1-\pi}{\pi} \frac{1}{g^{-1}(g\left( q \right)+\theta)'}=\frac{1-\pi}{\pi} \frac{g'(p)}{g'\left( q \right)}$$

Since tα has a closed form first derivative (Doebler & Holling, 2015), an explicit formula for c1 results by plugging in the derivative. Also note that the value of c1 depends on the prevalence, but when the same prevalence can be assumed for all studies, the prevalence factor (1-π) / π is the same for all studies. This means c1 values can be compared even when there is uncertainty about the prevalence. In this paper, we ignore the prevalence factor, so that

$$c_{1}= \frac{t_{\alpha}^{'}(p)}{t_{\alpha}^{'}(q)}$$

In order to assess the performance of models which did not report performance data we have used the extracted q-points from the ROC graphs. From the extracted q-points we calculated the log-DOR on which we performed a univariate analysis. The univariate analysis was performed on all models as well as the most important model per study. Forest plots were generated on the calculated log-DOR. The univariate analysis based on the log-DOR was also performed on the subgroups, both on all models and most important model per study within the subgroups.
