# Supplementary material for: Meta-analysis of diagnostic cell-free circulating microRNAs for breast cancer detection
Source: BMC Cancer. 2022 Jun 9;22:634. doi: 10.1186/s12885-022-09698-8 (PMC9178880; doi:10.1186/s12885-022-09698-8)
Supplement: Supplementary file 3 — Additional file 3: Supplementary Figure 1. SROCs of the subgroup bivariate models based on the most important model of each study. A) Plasma vs Serum B) Single vs Multiple panel miRNAs C) Endogenous v Exogenous normalizer D) With vs Without stage III and stage IV cases. Supplementary Figure 2. SROCs on miRNA-21-5p bivariate models. A) miRNA-21-5p SROC of all reported models. Points with the same colour in the graph represent models which come from the same study. B) miRNA-21-5p SROC of the most important model from each study. Supplementary Figure 3. Pooled estimates of sensitivity and specificity calculated on all models of studies stratified by year of publication. Linear regression was performed on both sensitivity and specificity across the years and no significant linear trend was observed. For both sensitivity and specificity, the linear regression estimates were around 0. [file 12885_2022_9698_MOESM3_ESM.pdf]

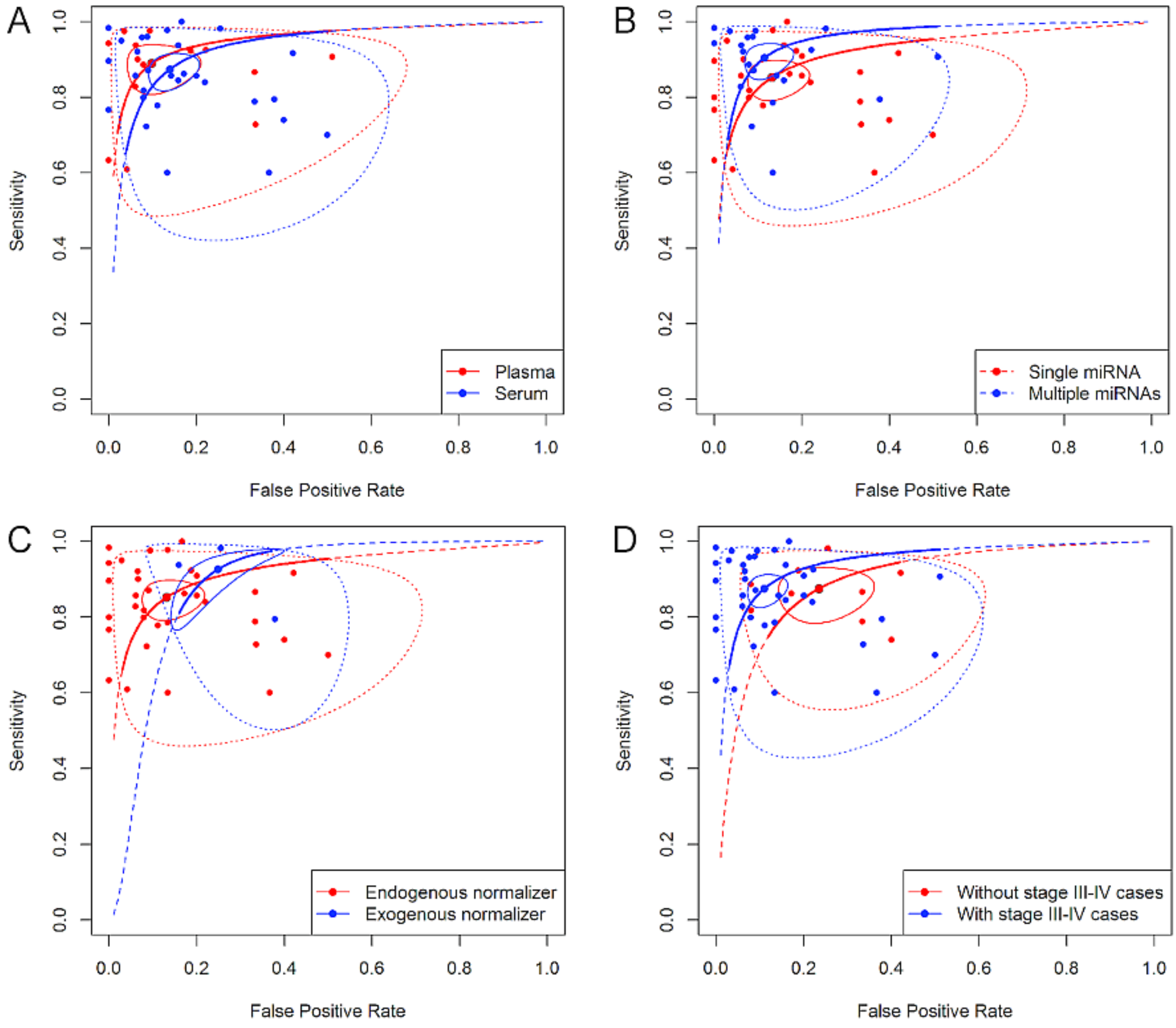

Supplementary Figure 1. SROCs of the subgroup bivariate models based on the most important model of each study **A)** Plasma vs Serum **B)** Single vs Multiple panel miRNAs **C)** Endogenous v Exogenous normalizer **D)** With vs Without stage III and stage IV cases.

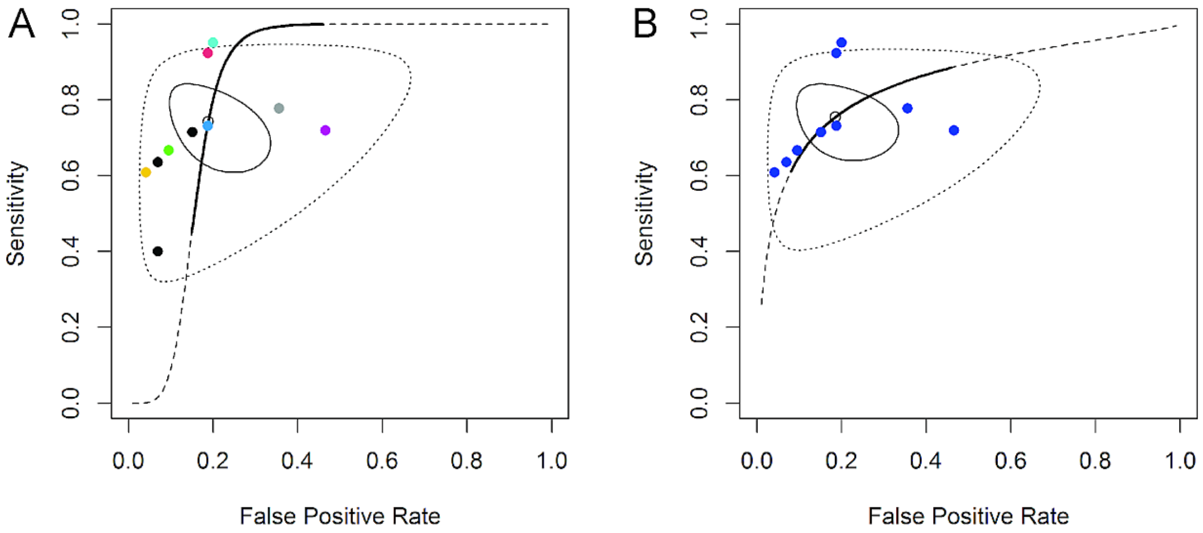

Supplementary Figure 2. SROCs on miRNA-21-5p bivariate models. **A)** miRNA-21-5p SROC of all reported models. Points with the same colour in the graph represent models which come from the same study. **B)** miRNA-21-5p SROC of the most important model from each study.

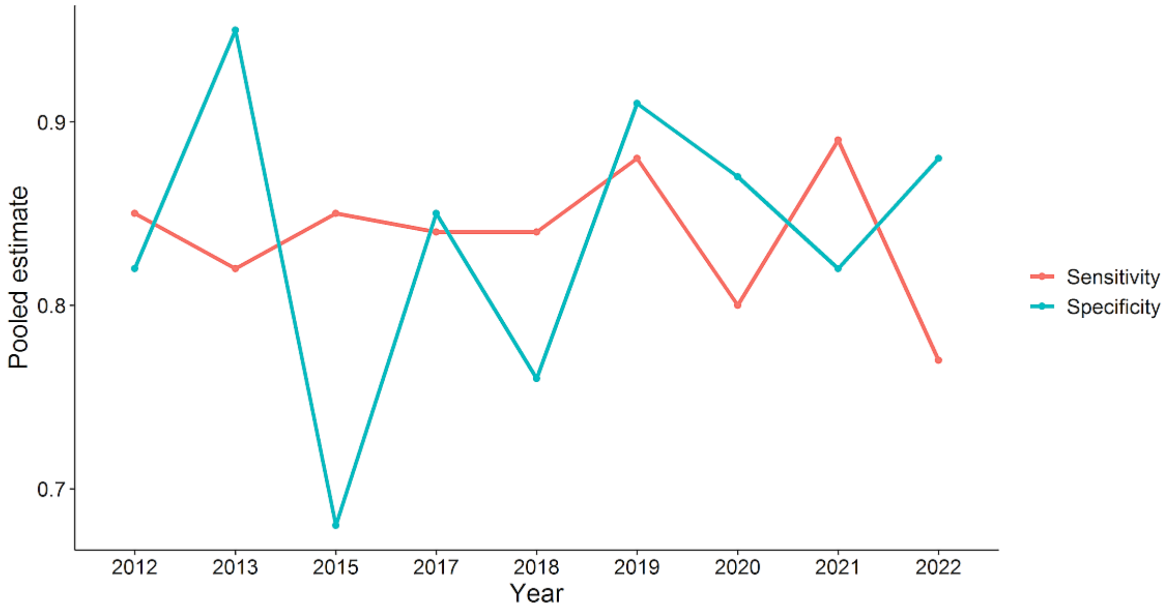

Supplementary Figure 3. Pooled estimates of sensitivity and specificity calculated on all models of studies stratified by year of publication. Linear regression was performed on both sensitivity and specificity across the years and no significant linear trend was observed. For both sensitivity and specificity, the linear regression estimates were around 0.
